# Effects of mulberry leaf enrichment with *Lepidium sativum* L. seed powder suspension on the economic parameters of *Bombyx mori* L

**DOI:** 10.1038/s41598-024-67128-0

**Published:** 2024-08-23

**Authors:** Ahlam A. Alfazairy, Deena A. Elsakhawy, Fatma A. El-Meniawi, Mohamed Hashem, Ibrahem A. Rawash

**Affiliations:** 1 Department of Applied Entomology and Zoology, Faculty of Agriculture, Alexandria University, Alexandria, Egypt; 2 Egyptian Plant Quarantine, Alexandria, Egypt

**Keywords:** *Bombyx mori*, *Lepidium sativum*, Cocoon parameters, Reproductive output, Biochemistry, Zoology

## Abstract

The phytochemicals of high nutritional and functional properties in *Lepidium sativum* L. (garden cress) seeds have nominated their seed powder (regardless of the concentration used) for enrichment of mulberry leaves in order to enhance *Bombyx mori* L. larval feeding, and consequently to gain ground in sericulture industry. As expected, *B. mori* larval feeding on *L. sativum-*enriched mulberry leaves showed not only a remarkable increase in mean values of certain economic parameters of *B. mori*, such as cocoon weight, cocoon shell weight, pupal weight, and egg yield, compared with the control group, but also showed a phenomenal increase in egg counts (on average, *ca.* 958–1256 eggs laid per female moth) and a significant increase in egg size (measured as egg surface area and egg volume). Male or female moth larval diet has significantly influenced the reproductive performance or fitness of both sexes of *B. mori* in terms of large-sized moths (measured as forewing, hind femur, and hind tibia lengths) and highly fecund moths (i.e., increased fecundity and spermatophore counts per female moth, and large-sized eggs). On the basis of *B. mori* female moth reproductive index, the female moths from *L. sativum*-fed larvae proved to have a lower reproductive index compared to their corresponding value for females of the control group, indicating more efficient utilization of larval resources for *B. mori* reproduction. Quantification of the three main physiological resources viz., protein, lipid and carbohydrate in the internal reproductive tract of *B. mori* female moths at death has nominated the female moth abdomens, or simply their bodies, as being a reasonable natural source of protein, lipid, and carbohydrate, to be involved in certain manufactures (e.g., pet feed formulations) instead of discarding them as a source of environmental pollution. Evidently, the *L. sativum* seed powder is of considerable interest because it remarkably improves the performance of such an economically important insect, *B. mori*. This is the first study for evaluating the efficacy of *L. sativum* seed powder in sericulture field to enhance *B. mori* productivity parameters.

## Introduction

The garden cress, *Lepidium sativum* is belonging to Family Brassicaceae. It has been considered as an important herbaceous-medicinal plant. Chemically, *L. sativum* seeds mainly contain protein, amino acids, fat or lipid, fatty acids, sterols (*β-*sitosterol), carbohydrate, reducing suger, fibres, mucilage, antioxidants (tocopherols; carotenoids), vitamins, minerals, phenolic compounds, steroids, cellulose, flavonoids, alkaloids, and other useful phytochemicals^1,2^.

Logically, such biochemical may represent the essential nutritional requirements for a healthy growth, development, performance, and the physiological functions of the mulberry silkworm, *Bombyx mori.* Comparatively, mulberry leaves, *Morus* spp. have been reported to contain the principle nutritional elements, water, protein, carbohydrate, fat, inorganic salts, minerals, and vitamins, for biological and productive parameters of *B. mori*^3,4^. In sericulture research work, to improve the economic parameters of *B. mori*, several considerable attempts have been carried out upon the enrichment of mulberry leaves with plant-based products^5,6^. As a monophagous and holometabolous insect, *B. mori* metabolism, growth, development, reproduction, and silk production mainly rely on its nutritional requirements and dietary supplementations. Hence, based on personal observations, the fine powder of *L. sativum* seeds, as a cheap and natural nutritive source, seems to be very essential for *B. mori* larval-, pupal-, and adult- performance. Additionally, *L. sativum*-seed powder functions as a phagostimulant, and antimicrobial agent^7^. Therefore, it seems fruitful to shed some light upon the economic gains of *L. sativum* seeds in sericulture field. In the literature, no available papers have been reported on the role of *L. sativum* seed powder in the field of sericulture; consequently, the objectives of the present study were (1) to highlight the economic benefit(s) of *B. mori* larval feeding, continuously or discontinuously, on *L. sativum*-enriched mulberry leaves on the pupal- and the adult-performance; (2) to quantify the total content of the three main physiological resources, protein, lipid, and carbohydrate, in the internal reproductive tract of *B. mori* female moths at death in order to digest to how extent could we make benefit from the female moth bodies after their death instead of discarding them as a source of environmental pollution.

## Materials and methods

### Rearing of *B. mori*

Eggs of *B. mori* (a local strain) were obtained from Sericulture Division, Plant Protection Institute, Ministry of Agriculture, Giza, Egypt, in March 2020. From the egg-hatching onward till the cocoon-spinning, *B. mori* larvae were reared on clean, soft and fresh mulberry leaves, *Morus alba* L. (treated or untreated leaves)—which collected in comply with relevant institutional, national, and international guidelines and legislation—under laboratory conditions of 24.62 ± 0.25 °C, 90.93 ± 0.43% R.H., and 14L:10D photoperiod. Rearing procedure was conducted in clean, sterilized, rectangular wooden trays (43 × 36 × 9 cm) with a wired base and top. Leafless branches of mulberry or casuarina (*Casuarina equisetifolia* L*.*) trees, and corrugated paper stripes were used for cocoon spinning. Regular cleaning of rearing trays was carried out.

### Effect of enrichment of mulberry leaves with *L. sativum* seed powder on some economic parameters of *B. mori*

*L. sativum* seeds used in this study were obtained from the local market of the type grown in Al-Qaseem area in Saudi Arabia, and authenticated by an expert taxonomist. The seeds were ground by an electric grinder to get a fine powder which was stored in an airtight glass jar. Three concentrations (0.25, 0.50, and 1.00%, w/v) were prepared in distilled water. Based on the tested concentration, the calibrated weight of *L. sativum* seed powder and distilled water were thoroughly dissolved in a suitable container until getting a sort of a homogenous suspension. Fresh and clean mulberry leaves were dipped, separately, in the tested concentration for *ca.* 1 min (with stirring in order to ensure a complete coverage of the tested suspension for the leaf-upper and lower-surface) and air-dried for 5–10 min.

*B. mori* larvae were allocated into three groups; the first group served as the control, the second and the third groups served as the treatments. Control larvae were fed (from egg hatching till cocoon spinning) on mulberry leaves treated with distilled water only. Treatment-larvae of the second group were fed (from egg hatching till cocoon spinning) continuously on mulberry leaves enriched with *L. sativum* seed powder suspension of each tested concentration (continuous feeding); whereas *B. mori* larvae of the third group were fed on treated leaves for 48-h interval, then untreated mulberry leaves were supplied for 48-h interval; then treated mulberry leaves were supplied for another 48-h interval, and so on till cocoon spinning (discontinuous feeding). All the treatments and the control were replicated thrice, and each replication consisted of 100 larvae.

The observations were recorded on the following economic and physiological parameters: cocoon weight, cocoon shell weight, cocoon shell ratio percentage, pupal weight, adult longevity, female moth fecundity (number of deposited eggs per female), number of spermatophores per female moth, and egg size (i.e., egg length and width; egg surface area and volume). Egg volume was estimated as a prolate spheroid, using the formula V = 4/3 π abc (a;b;c are the radii at different planes^8^). Dimensions of the egg (a, b, c) were measured using an ocular micrometer on a dissecting binocular microscope (20X). The egg surface area was calculated using the formula of an ellipse (A = π ab; where a = length of semimajor axis and b = length of semiminor axis^9^). Also, the effect of body size of male and female moths, and number of matings (number of spermatophores per female) on female moth reproductive output (fecundity and longevity) were evaluated. The lengths of forewing, hind femur, or hind tibia (as indices of body size) were measured using both of a millimeter paper for scale and a dissecting binocular microscope at 20X^10,11^. The total protein-, lipid- and carbohydrate-content of female moth internal reproductive tract (*ca.* 60 females per treatment) was also quantified directly after their death. The total protein, lipid, and carbohydrate contents were estimated from frozen samples (− 20 °C) of the female moth internal reproductive organ homogenates by using the Kjeldahl method and Soxhlet^12,13^.

### Statistical analysis

Results were statistically analyzed using the ANOVA followed by Duncan’s multiple range test at 5% probability level. The results were represented as Mean ± SE. Statistical analyses were performed using the IBM SPSS Statistics for Windows, Version 20, Armank, NY, IBM Crop, 2011.

## Results

### Performance of *B. mori *in the presence of *L. sativum* seed powder in its larval diet

The effects of enrichment of mulberry leaves with *L. sativum* seed powder on *B. mori* performance (pupal and adult parameters) are summarized in Tables 1, 2, 3, 4, 5, 6 and 7.

**Table 1:** Effect of continuous (c) or discontinuous (d) feeding of *B. mori* larvae on mulberry leaves enriched with the indicated concentrations of *L. sativum* seed powder on certain economic parameters of their cocoons*.

Cocoon economic parameter	Mean values (gm) ± S.E. at indicated concentrations (%) of *L. sativum* seed powder (% change over control)**							*F*_df_; *P*
	0.00%	0.25%		0.50%		1.00%		
	(Control)	Larval feeding		Larval feeding		Larval feeding		
		Continuous	Discontinuous	Continuous	Discontinuous	Continuous	Discontinuous	
Cocoon weight*** (gm)	1.134^d^ ± 0.013	1.571^a^ ± 0.020 (38.53)	1.473^b^ ± 0.028 (29.90)	1.565^a^ ± 0.022 (38.03)	1.528^ab^ ± 0.017 (34.75)	1.567^a^ ± 0.021 (38.21)	1.410^c^ ± 0.017 (24.39)	60.425_6,896_; 0.000
Pupal weight (gm)	0.813^d^ ± 0.008	1.204^ab^ ± 0.015 (48.06)	1.121^c^ ± 0.022 (37.94)	1.217^a^ ± 0.015 (49.74)	1.173^b^ ± 0.012 (44.31)	1.227^a^ ± 0.013 (50.97)	1.091^c^ ± 0.012 (34.15)	99.279_6,896_; 0.000
Cocoon shell weight (gm)	0.321^c^ ± 0.005	0.367^a^ ± 0.005 (14.37)	0.351^ab^ ± 0.006 (9.54)	0.348^b^ ± 0.008 (8.35)	0.355^ab^ ± 0.005 (10.54)	0.340^b^ ± 0.084 (5.92)	0.320^c^ ± 0.005 (− 0.34)	8.059_6,896_; 0.000
Cocoon shell ratio (%)	28.183^a^ ± 0.115	23.315^c^ ± 0.05 (− 17.27)	23.877^b^ ± 0.087 (− 15.28)	21.965^e^ ± 0.224 (− 22.06)	23.151^c^ ± 0.084 (− 17.86)	21.380f. ± 0.218 (− 24.14)	22.542^d^ ± 0.107 (− 20.02)	251.158_6,896_; 0.000

Values followed by different letters on the same row are significantly different at α level = 0.05; ANOVA followed by Duncan’s multiple range test.

*Sample size (n) = 129.

**Positive or negative values in parentheses represent the percent increase or decrease, respectively, over the control.

***7-days post cocoon spinning.

**Table 2:** Comparison of spermatophore count (no. of matings) to egg count laid by *B. mori*-mated female moths* just after their death at indicated treatments.

*B. mori* moth parameter	Larval treatment at *L. sativum* indicated conc. (%)							*F*_df_; *P*
	0.00%	0.25%		0.50%		1.00%		
	Control	Continuous feeding	Discontinuous feeding	Continuous feeding	Discontinuous feeding	Continuous feeding	Discontinuous feeding	
Mean no. of spermatophores per female ± S.E. (% change over control)**	1.37^d^ ± 0.05	2.99^a^ ± 0.15 (117.47)	3.00^a^ ± 0.12 (118.42)	2.57^bc^ ± 0.16 (86.76)	2.46^c^ ± 0.13 (79.38)	2.26^c^ ± 0.15 (64.54)	2.89^ab^ ± 0.10 (110.10)	25.690_6,448_; 0.000
Mean no. of eggs laid per female ± S.E. (Fecundity) (% change over control)**	450.63^d^ ± 17.38	731.16^c^ ± 24.24 (62.26)	887.83^b^ ± 51.89 (97.02)	1267.19^a^ ± 72.52 (181.21)	1364.79^a^ ± 43.46 (202.87)	1250.14^a^ ± 78.65 (177.42)	1354.04^a^ ± 49.63 (200.48)	68.941_6,385_; 0.000

Values followed by different letters in a column are significantly different at α level = 0.05; ANOVA followed by Duncan’s multiple range test.

*Sample size (n) ranged between 42- and 83-individual.

**Values in parentheses represent the percent increase over the control.

**Table 3:** Effect of the indicated treatments of the larval continuous or discontinuous feeding on mulberry leaves enriched with the indicated concentrations of *L. sativum*-seed powder suspensions on certain parameters of *B. mori* moths.

*B. mori* moth parameter		Calculated value	Treatment at *L. sativum* indicated conc. (%)							*F*_df_; *P*
			0.0%	0.25%		0.50%		1.00%		
			(Control)	Larval feeding		Larval feeding		Larval feeding		
				Continuous	Discontinuous	Continuous	Discontinuous	Continuous	Discontinuous	
Longevity (days)	Female n = 47–72	Mean ± S.E. (% change over control)*	7.19^a^ ± 0.35	7.79^a^ ± 0.28 (8.22)	7.82^a^ ± 0.31 (8.73)	7.38^a^ ± 0.41 (2.62)	7.90^a^ ± 0.29 (9.81)	7.45^a^ ± 0.39 (3.57)	7.35^a^ ± 0.34 (2.22)	0.718_6,430_;0.635
	Male n = 47–72	Mean ± S.E. (% change over control)*	7.28^b^ ± 0.34	9.61^a^ ± 0.32 (32.10)	10.15^a^ ± 0.36 (39.40)	8.17^b^ ± 0.53 (12.26)	7.99^b^ ± 0.39 (9.73)	9.39^a^ ± 0.44 (29.05)	9.60^a^ ± 0.41 (31.91)	7.747_6,430_;0.000
Body size index: Forewing length (mm)	Female n = 84–144	Mean ± S.E. (% change over control)*	17.79^e^ ± 0.09	18.80^d^ ± 0.10 (5.67)	20.59^c^ ± 0.20 (15.72)	21.90^b^ ± 0.14 (23.13)	22.79^a^ ± 0.12 (28.11)	21.70^b^ ± 0.19 (22.00)	22.51^a^ ± 0.12 (26.56)	248.928_6,784_;0.000
	Male n = 41–87	Mean ± S.E. (% change over control)*	16.60^c^ ± 0.19	18.26^a^ ± 0.16 (10.02)	18.57^a^ ± 016 (11.88)	18.56^a^ ± 0.30 (11.80)	18.19^a^ ± 0.15 (9.58)	17.44^b^ ± 0.25 (5.07)	18.21^a^ ± 0.16 (9.73)	16.609_6,403_;0.000
Hind femur length (mm)	Female n = 84–144	Mean ± S.E. (% change over control)*	3.44f. ± 0.01	3.51^e^ ± 0.01 (2.06)	3.61^d^ ± 0.02 (4.90)	3.73^c^ ± 0.01 (8.31)	3.90^a^ ± 0.01 (13.27)	3.75^c^ ± 0.02 (8.98)	3.82^b^ ± 0.01 (11.0)	169.698_6,784_;0.000
	Male n = 41–87	Mean ± S.E. (% change over control)*	3.41^d^ ± 0.02	3.67^a^ ± 0.02 (7.60)	3.60^ab^ ± 0.02 (5.49)	3.60^ab^ ± 0.03 (5.44)	3.61^ab^ ± 0.02 (5.71)	3.49^ cd^ ± 0.04 (2.15)	3.54^bc^ ± 0.03 (3.57)	12.699_6,403_;0.000
Hind tibia length (mm)	Female n = 84–144	Mean ± S.E. (% change over control)*	3.32f. ± 0.01	3.41^e^ ± 0.01 (2.76)	3.50^d^ ± 0.02 (5.59)	3.61^c^ ± 0.01 (8.83)	3.83^a^ ± 0.01 (15.46)	3.61^c^ ± 0.02 (8.90)	3.73^b^ ± 0.02 (12.34)	157.667_6,784_;0.000
	Male n = 41–87	Mean ± S.E. (% change over control)*	3.31^c^ ± 0.02	3.58^a^ ± 0.02 (8.22)	3.55^ab^ ± 0.03 (7.19)	3.50^ab^ ± 0.02 (5.53)	3.54^ab^ ± 0.02 (7.03)	3.47^b^ ± 0.03 (4.92)	3.48^ab^ ± 0.03 (5.27)	16.288_6,403_;0.000
Female moth weight** (gm)	n = 49–84	Mean ± S.E. (% change over control)*	0.34^ab^ ± 0.02	0.30^b^ ± 0.02 (− 13.25)	0.30^b^ ± 0.02 (− 12.82)	0.33^b^ ± 0.02 (− 3.69)	0.30^b^ ± 0.01 (− 20.08)	0.41^a^ ± 0.03 (18.08)	0.28^b^ ± 0.02 (− 19.76)	3.372_6,444_;0.003
Egg size index: Egg length	n = 100	Mean ± S.E. (% change over control)*	1.41^d^ ± 0.007	1.51^c^ ± 0.003 (6.87)	1.50^c^ ± 0.003 (6.48)	1.54^ab^ ± 0.004 (8.97)	1.51^c^ ± 0.003 (7.16)	1.55^a^ ± 0.003 (9.5)	1.53^b^ ± 0.003 (8.29)	122.872_6,693_;0.000
Egg width	n = 100	Mean ± S.E. (% change over control)*	1.05^d^ ± 0.006	1.18^c^ ± 0.006 (12.35)	1.17^c^ ± 0.005 (11.44)	1.21^b^ ± 0.006 (15.35)	1.20^b^ ± 0.005 (14.35)	1.26^a^ ± 0.003 (19.78)	1.25^a^ ± 0.004 (18.97)	190.854_6,693_;0.000
Egg surface area (mm^2^)	n = 100	Mean ± S.E. (% change over control)*	1.164^e^ ± 0.009	1.396^d^ ± 0.008 (20.01)	1.380^d^ ± 0.007 (18.61)	1.462^b^ ± 0.008 (25.66)	1.425^c^ ± 0.007 (22.48)	1.525^a^ ± 0.005 (31.07)	1.499^a^ ± 0.007 (28.79)	256.825_6,693_;0.000
Egg volume (mm^3^)	n = 100	Mean ± S.E. (% change over control)*	0.810^e^ ± 0.011	1.098^d^ ± 0.013 (35.52)	1.082^d^ ± 0.013 (33.55)	1.205^b^ ± 0.014 (48.73)	1.149^c^ ± 0.011 (41.86)	1.308^a^ ± 0.008 (61.51)	1.270^a^ ± 0.010 (56.75)	196.797_,693_;0.000

Values followed by different letters on the same row are significantly different at α level = 0.05; ANOVA followed by Duncan’s multiple range test.

*Positive or negative values in parentheses represent the percent increase or decrease, respectively, over the control.

**Measured immediately after the moth death.

**Table 4:** Summary of the outcome of the present treatments addressing the effect of enrichment of mulberry leaves with* L. sativum* seed powder on certain *B. mori*—pupal economic parameters.

Pupal parameter	Calculated values of the percent change (increase) over control*					
	Treatment					
	0.25%		0.50%		1.00%	
	Larval feeding		Larval feeding		Larval feeding	
	Continuous	Discontinuous	Continuous	Discontinuous	Continuous	Discontinuous
Cocoon weight (gm)	38.5	29.9	38.0	34.8	38.2	24.4
Pupal weight (gm)	48.1	37.9	49.7	44.3	51.0	34.2
Cocoon shell weight (gm)	14.4	9.5	8.4	10.5	5.9	− 0.34**

*% change (increase) over control = treatment − control/control × 100.

**A marginally non–significant decrease over the control.

**Table 5:** Summary of the outcome of the present treatments addressing the effect of enrichment of mulberry leaves with *L. sativum* seed powder on certain *B. mori*—moth fitness parameters.

*B. mori* moth parameter	Calculated values of the percent change (increase) over the control* (%)											
	Treatment											
	0.25%				0.50%				1.00%			
	Larval feeding				Larval feeding				Larval feeding			
	Continuous		Discontinuous		Continuous		Discontinuous		Continuous		Discontinuous	
Egg production (fecundity)	62.3		97.0		181.2		202.9		177.4		200.5	
Spermatophore counts per female	117.5		118.4		86.8		79.4		64.5		110.1	
Longevity	Female	Male	Female	Male	Female	Male	Female	Male	Female	Male	Female	Male
	8.2	32.1	8.7	39.4	2.6	12.3	9.8	9.7	3.6	29.1	2.2	31.9
Body size index												
Forewing length	5.7	10.0	15.7	11.9	23.1	11.8	28.1	9.6	22.0	5.1	26.6	9.7
Hind femur length	2.1	7.6	4.9	5.5	8.3	5.4	13.3	5.7	9.0	2.2	11.0	3.6
Hind tibia length	2.8	8.2	5.6	7.2	8.8	5.5	15.5	7.0	8.9	4.9	12.3	5.3
Egg size index												
Length	6.9		6.5		9.0		7.2		9.5		8.3	
Width	12.4		11.4		15.4		14.4		19.8		19.0	
Surface area	20.0		18.6		25.7		22.5		31.1		28.8	
Volume	35.5		33.6		48.7		41.9		61.5		56.8	
Reproductive index	0.00165		0.00126		0.00096		0.00086		0.00098		0.00081	

*% change (increase) over control = treatment − control/control × 100.

**Table 6:** The physiological cost(s) of being a highly fecund-*B. mori* female moths based on the quantified physiological resources, protein-, lipid-, and carbohydrate-content (mg/gm) in their internal reproductive tract at death.

The quantified parameter* in *B. mori* female moth internal reproductive tract (mg/gm)	Calculated value	Treatment at *L. sativum* indicated conc. (%)							*F*_df_; *P*
		0.00%	0.25%		0.50%		1.00%		
		(Control)	Larval feeding		Larval feeding		Larval feeding		
			Continuous	Discontinuous	Continuous	Discontinuous	Continuous	Discontinuous	
Total protein content	(% change over control)**	61.62^a^ ± 0.06	58.90^d^ ± 0.01	58.69^d^ ± 0.17	60.27^c^ ± 0.19	60.41^c^ ± 0.05	61.14^ab^ ± 0.13	60.51^bc^ ± 0.27	51.825_6,14_;0.000
			(− 4.42)	(− 4.76)	(− 2.20)	(− 1.96)	(− 0.78)	(− 1.80)	
Total lipid content	Mean ± S.E. (% change over control)**	22.92^d^ ± 0.23	26.48^ab^ ± 0.05	24.07^c^ ± 0.30	23.39^d^ ± 0.03	26.04^b^ ± 0.01	17.76^e^ ± 0.15	26.85^a^ ± 0.10	386.859_6,14_;0.000
			(15.53)	(5.00)	(2.05)	(13.60)	(− 22.51)	(17.13)	
Total carbohydrate content	Mean ± S.E. (% change over control)**	0.74^c^ ± 0.17	0.11^d^ ± 0.04	1.52^b^ ± 0.13	0.85^c^ ± 0.22	0.10^d^ ± 0.04	6.34^a^ ± 0.02	0.58^c^ ± 0.16	270.222_6,14_;0.000
			(− 85.58)	(105.41)	(14.42)	(− 86.93)	(756.31)	(− 22.07)	
Mean no. of eggs laid per female (fecundity)	Mean ± S.E. (% change over control)**	450.63^d^ ± 17.38	731.16^c^ ± 24.24	887.83^b^ ± 51.89	1267.19^a^ ± 72.52	1364.79^a^ ± 43.46	1250.14^a^ ± 78.65	1354.04^a^ ± 49.63	68.941_6,385_ ; 0.000
			(62.26)	(97.02)	(181.21)	(202.87)	(177.42)	(200.48)	

Values followed by different letters on the same row are significantly different at α level = 0.05; ANOVA followed by Duncan’s multiple range test.

*Measured immediately after female moth death.

**Positive or negative values in parentheses represent the percent increase or decrease, respectively, over the control.

**Table 7:** Summary of the outcome of the present treatments of *B. mori* larval feeding, continuously (c) or discontinuously (d), on mulberry leaves enriched with the garden cress (*L. sativum*) seed powder (regardless of the concentration used) on some desired parameters in sericulture.

Parameter	Mean* ± S.E			% change over control		Parameter	Mean* ± S.E			% change over control	
	Control	Treatment		Treatment			Control	Treatment		Treatment	
		c	d	c	d			c	d	c	d
Pupal weight (gm)	0.813^c^ ± 0.008	1.216^a^ ± 0.014	1.128^b^ ± 0.015	49.59	38.80	Total protein content**	61.620^a^ ± 0.051	60.100^b^ ± 0.298	59.869^b^ ± 0.277	− 2.47	− 2.84
Cocoon shell weight (gm)	0.321^b^ ± 0.005	0.351^a^ ± 0.007	0.342^a^ ± 0.005	9.54	6.58	Total lipid content**	22.920^b^ ± 0.078	22.543^b^ ± 1.142	25.650^a^ ± 0.378	− 1.65	11.91
Cocoon weight (gm)	1.134^c^ ± 0.013	1.567^a^ ± 0.021	1.470^b^ ± 0.020	38.26	29.68	Total carbohydrate content**	0.740^b^ ± 0.061	2.430^a^ ± 0.881	0.731^b^ ± 0.195	228.38	− 1.22
Cocoon shell ratio (%)	28.183^a^ ± 0.115	22.220^c^ ± 0.138	23.190^b^ ± 0.067	− 21.16	− 17.72	Female moth body weight at death (gm)	0.344^a^ ± 0.020	0.332^ab^ ± 0.013	0.281^b^ ± 0.010	− 3.46	− 18.31
Female fecundity***	450.625^c^ ± 17.375	958.412^b^ ± 33.083	1255.924^a^ ± 34.194	112.69	178.71	Body size index: Female forewing length (mm)	17.788^c^ ± 0.088	20.135^b^ ± 0.113	22.273^a^ ± 0.095	13.19	25.21
Female longevity (days)	7.194^a^ ± 0.347	7.757^a^ ± 0.243	7.786^a^ ± 0.176	7.82	8.22	Female hind femur length (mm)	3.443^c^ ± 0.010	3.629^b^ ± 0.013	3.817^a^ ± 0.010	5.40	10.85
Male longevity (days)	7.282^b^ ± 0.327	9.466^a^ ± 0.264	9.364^a^ ± 0.316	29.99	28.58	Female hind tibia length (mm)	3.319^c^ ± 0.014	3.504^b^ ± 0.010	3.730^a^ ± 0.011	5.59	12.38
Egg surface area (mm^2^)	1.164^c^ ± 0.009	1.461^a^ ± 0.004	1.435^b^ ± 0.004	25.58	23.29	Male forewing length (mm)	16.598^b^ ± 0.188	18.205^a^ ± 0.143	18.311^a^ ± 0.090	9.68	10.32
Egg volume (mm^3^)	0.810^c^ ± 0.011	1.204^a^ ± 0.006	1.167^b^ ± 0.007	48.60	44.06	Male hind femur length (mm)	3.414^b^ ± 0.091	3.614^a^ ± 0.019	3.591^a^ ± 0.014	5.84	5.16
Spermatophore count per female	1.374^b^ ± 0.053	2.740^a^ ± 0.119	2.800^a^ ± 0.090	99.51	103.86	Male hind tibia length (mm)	3.308^b^ ± 0.023	3.529^a^ ± 0.018	3.525^a^ ± 0.014	6.69	6.56

*Values followed by different letters on the same row are significantly different at α level = 0.05; ANOVA followed by Duncan’s multiple range test.

**The total protein, lipid or carbohydrate content (mg/gm) in female moth internal reproductive tract at death.

***Number of eggs laid per female.

### Pupal economic parameters

As shown in Tables 1 and 4, the recorded significant increases in cocoon parameters, cocoon weight, pupal weight, and cocoon shell weight, at all indicated treatments were remarkably better than the control groups. The percent increase values over their control values ranged approximately between 24.4–38.5%, 34.2–51.0%, and 5.9–14.4%, respectively. Only a marginal decrease (− 0.34%), but statistically not significant, was recorded for the cocoon shell weight obtained from cocoons produced by *B. mori* larvae fed, discontinuously, on mulberry leaves enriched with a 1% *L. sativum* (0.320 ± 0.005 gm) when compared to the control group (0.321 ± 0.005 gm) (Table 1). On the other hand, the cocoon shell ratio (%) in the control group was significantly higher than that of the *L. sativum*-fed group. The reduction ranged, nearly, from 15.3% to 24.1% (Table 1). This reduction could be attributed to the positive increase of pupal weights in the experimental groups (Tables 1, 4) as compared to the significantly low pupal weights in the control group. In general, no obvious trend was observed in this study to address the effect of both the *L. sativum*-concentration used and the experimental larval feeding pattern (i.e., continuous or discontinuous) on the studied cocoon parameters. Meanwhile, higher mean values of both the cocoon weight and the pupal weight were evident when *B. mori* larvae were fed continuously on mulberry leaves enriched with *L. sativum*; irrespective of *L. sativum*-concentration used (Tables 1, 4).

### Adult economic parameters

#### Fecundity (no. of eggs laid per female)

As shown in Tables 2 and 5, *B. mori* larval feeding, continuously or discontinuously, on *L. sativum*-enriched mulberry leaves leads to a significant increases in fecundity by nearly two to threefold or 62.26–202.87% in the experimental groups when compared to the control group. Evidently, the maximum increases of 177.42–202.87% in fecundity or the egg yield averages (1250.14 ± 78.65–1364.79 ± 43.46 egg) were accompanied with *L. sativum*-concentration higher than 0.25% (i.e., 0.50 or 1.00%). It appears, to a large extent that irrespective of the *L. sativum*-tested concentration, low or high, the highest egg yield averages were recorded in *B. mori-* discontinuous larval feeding pattern of the experimental group (Tables 2, 5).

#### Egg size

As an index of egg size, the egg length, width, volume, and surface area were estimated. *B. mori* larval feeding on* L. sativum*-enriched mulberry leaves considerably affected the estimated egg parameters. In the experimental group, mean values of these parameters were significantly increased when compared to the control group (Tables 3, 5). On calculating the percent increase values over their control ones, a minimal increase of 6.5% and a maximal one of 9.5% were recorded in the egg length; whereas the corresponding values for the egg width were 11.4% and 17.8% (Table 5). Also, the egg surface area and volume were increased, *ca.,* by 33.65–61.5% and 18.6–31.1%, respectively (Tables 3, 5). Such an obvious percent increase in the egg size parameters over the control was, in general, a conc.-dependent and a larval feeding pattern-dependent as well. In other words, higher increases were always associated with both of higher concentrations of *L. sativum* seed powder and the larval continuous feeding pattern.

#### Male or female moth body size

The lengths of forewing, hind femur, and hind tibia were measured as an index of *B. mori* male or female moth body size. As shown in Tables 3 and 5, the average body size of the male or the female, was significantly increased. The forewing mean lengths were increased by up to 5.07–11.88% or 5.67–28.11% in the male or the female moths, in respect (Table 3). Among the calculated mean values of female or male hind femur length, the percent increase, in the treatments, over the control had ranged from 2.06 to 13.27% in female moths, or ranged from 2.15 to 7.60% in male moths (Table 3).

Also, when the female or male moth body size was measured as a hind tibia length, approximately similar increases in the mean values of hind tibia lengths occurred by up to 2.76–15.46% in the female, or 4.92–8.22% in the male (Table 3). As is evident from the data presented in Tables 3 and 5, the body size indices of both sexes were, in general, significantly increased in length in a conc.-dependent way, and a larval feeding pattern-dependent as well; where, in general, it appears that the observed increase in the above-mentioned indices was associated with *B. mori-*discontinuous larval feeding on *L. sativum*- enriched mulberry leaves. However, few exceptions did not follow such a dependency (Tables 3, 5).

#### Longevity of female and male moths

In the experimental group, the female moth longevity recorded an increase of 2.22–9.81%; however, this increase was not significantly different to that of the control group. Also, an increase of 9.73–39.40% was recorded for the male moth longevity, in the treatments; but such increases were significantly different to that of the control except for the male longevity at conc. 0.50% (Table 3). In terms of male or female moth longevity, such increases, significant or insignificant, seemed to be neither a dose–dependent nor a larval feeding pattern-dependent (Table 3).

#### Relationship between spermatophore counts and egg counts

In terms of spermatophore counts in the bursa copulatrix of *B. mori-*mated females, the number of matings by their males ranged from one to two (mean value = 1.37) in the control group; whereas the corresponding number of matings in the experimental group ranged from one to six (mean values = 2.26–3.00), but never mate more than six times. The differences in mean spermatophore numbers between the two groups, the control and the experimental, were significant in favour of the latter group (Table 2).

Based on the calculated values of the percent increase, in the experimental group, over that of the control one, the mean spermatophore counts per female showed to be increased by up to 64.54–118.42% (Table 2). Also, the results show that in the control group, 62.65% or 37.35% of *B. mori* males were able to mate once or twice, respectively. In the experimental group, at conc. 0.25% and a continuous larval feeding pattern, 3.90, 24.68, 24.68, 29.87, and 10.39% of males could achieve one, two, three, four, and five matings, respectively; whereas with the discontinuous larval feeding pattern, the corresponding percentages of one, two, three, four, and five matings were, in respect, 1.67, 31.67, 38.33, 21.67, and 6.67%. At the tested conc. of 0.50 or 1.00%, whether at the continuous or the discontinuous larval feeding pattern, the number of matings for males ranged from one to four. Only one male (1.45%) was able to achieve six matings. The maximum mean number of spermatophores per female (*ca.,* 3 spermatophores) was observed in both patterns of the larval feeding, continuous and discontinuous, at the three tested concentrations of *L. sativum* seed powder; while it was minimum (*ca.*, 2.3 spermatophores) at the continuous larval feeding pattern with conc. 1.00% (Table 2). As shown in Table 2, the mean spermatophore counts were remarkably increased in the experimental group when compared to that of the control group; such an increase seems to be neither dose- dependent nor larval feeding pattern, continuous or discontinuous.

Also, the findings in Table 2 reveal that, in terms of both the average spermatophore counts and the average egg counts, *B. mori* female moths that mated once (averagely1.37-time) lay significantly fewer eggs (mean = 450.63 eggs; in the control group) than the females mated averagely from 2 to 3 times (mean = 731.16–1364.79 eggs; in the experimental group). Thus, the increase in spermatophore numbers increases the average egg yield or fecundity of *B. mori* female moth by up to 2–3 times (62.26–202.87%) when compared to that of the control group. Therefore, it seems that *B. mori* female fecundity is dependent upon mating frequency.

#### Reproductive index

In general, *L. sativum* seed powder yielded large pupae and highly fecund moths (i.e., females or males) that laid profuse and large-sized eggs or mated more than twice compared to that of the controls (Table 2). *B. mori* adult females obtained from the untreated mulberry leaves-fed larvae (the controls) had the highest reproductive index (0.00180), revealing less efficiency in utilizing larval nutritive resources for reproduction (i.e., low mean values for egg count and size; Tables 2, 5). The reproductive index was estimated by dividing the mean value of the pupal weight (gm) by the corresponding value of the fecundity (no. of eggs laid per female). The enrichment of mulberry leaves with *L. sativum* seed powder led to lower values for the reproductive indices, ranged from 0.00080 to 0.00165, indicating better efficiency in utilization of larval resources for reproduction (Tables 2, 5). In terms of the reproductive index, it seems that *B. mori* female moths which having low indices, as in the experimental group, lay significantly large eggs; while those having a higher index, as in the control group, lay significantly small ones (Tables 3, 5).

#### Gain that could be benefited from the key physiological resources, protein-, lipid-, and carbohydrate-content, in *B. mori* female moths at death

Based on the present findings, it was fruitful to shed some light upon the fate of *B. mori* female moths after achieving their job in sericulture field, viz., egg production performance. In other words, what is the added value of *B. mori* female moths after their death in order not to be an addable burden from the environmental pollution point of view; plus loss of important nutrients such as, proteins, lipids, and carbohydrates. According to the literature review, all researches which are related to this subject have been carried out on determination of the above-mentioned reserves, as valuable nutritional resources, in *B. mori* pupae. However, less attention (i.e., no study) has been paid to the total protein-, lipid-, or carbohydrate-content in *B. mori* female moths after their death. Therefore, the purpose of this experiment was to quantify the total protein-, lipid- and carbohydrate-content in the internal reproductive tract of *B. mori* female moths at death; hopefully to add value(s) besides their main performance, the egg production (e.g., providing certain manufactures with natural raw materials, e.g., pet feed formulations, from the moth abdomens, where the internal reproductive organs occupy most of the abdomen). Table 6 summarizes the relationship between the total protein-, lipid-, and carbohydrate-content, in the internal reproductive tract of *B. mori* female moths at death and egg production (fecundity) at indicated treatments.

#### The total protein content

The average values (± S.E.) of the total protein content (mg/gm) at the indicated treatments recorded a marginally significant decrease of about 0.80–4.80% over the control, except for the corresponding value (61.14 ± 0.13 mg/gm) at both the highest conc. used (1.00%) and the continuous larval feeding pattern, where a marginal and statistically insignificant decrease (0.78%) over the control mean value (61.62 ± 0.06 mg/gm) was recorded. In the experimental group, the quantified mean values of the total protein content in the internal reproductive tract of *B. mori* female moths at death ranged from 58.69 to 61.14 mg/gm. As might be expected for a protein content to be decreased after the egg production performance; however, the relatively marginal reduction in the average protein content, in the experimental group, was greatly associated with remarkable average numbers of eggs laid per female which ranged between 731.16 ± 24.24 eggs and 1364.79 ± 43.46 eggs, as compared to the significantly lower corresponding value of 450.63 ± 17.38 eggs in the control group (Table 6). Hence, it seems logical that the presence of *L. sativum* seed powder, in *B. mori* larval diet, would nutritionally have a strong physiological effect on both egg yield and egg size, towards yielding optimal egg counts and egg size (see Tables 3, 5, 6). Also, the decrease in the total protein content in the internal reproductive tract of *B. mori* female moths appeared to be a dose-dependent, and apt to be the least towards higher concentrations (i.e., > 0.25%). There was no statistically significant difference in protein content mean values at continuous and discontinuous larval feeding patterns (Table 6).

#### The total lipid content

The total lipid content in the internal reproductive tract of *B. mori* female moths, at death, was significantly higher in the experimental group than those in the control group by 2.05–17.13%, except for the corresponding value (17.76 ± 0.15 mg/gm) at both the highest conc. used (1.00%) and the continuous larval feeding pattern; where a significant decrease (22.51%) over the control mean value (± S.E.) (22.92 ± 0.23 mg/gm) was observed (Table 6). Also, data in Table 6 show that, in general, the mean value of the total lipid content was increased in a dose-dependent way. In general, the quantified significant increase of lipid content, that ranged from 17.76 ± 0.15 mg/gm to 26.85 ± 0.10 mg/gm, must ultimately be resulted from a well-enriched larval diet, since the *B. mori* female moths do not feed. That seems logical where *B. mori* female moths obtained from larvae fed on more nutritious mulberry leaves (i.e., enriched with *L. sativum* seed powder) carried over, in general, more lipid reserves from the larval stage than those obtained from larvae reared on a less nutritious diet (i.e., the control group). Moreover, as shown in Table 6, *B. mori* larvae reared on a nutrient-rich diet (the experimental group) gave rise to female moths which laid remarkably more eggs than those emerged from larvae reared on a less nutritious one (the control group).

#### The total carbohydrate content

The highest total carbohydrate content (6.34 ± 0.02 mg/gm) in the internal reproductive tract of *B. mori* female moths at death was detected at the continuous larval feeding pattern with *L. sativum* seed powder of conc. 1.00%. This increase, by *ca*. 756%, was significant as compared to the corresponding mean value (± S.E.) in the control group (0.74 ± 0.17 mg/gm); followed by an increase of *ca*. 105% (1.52 ± 0.13 mg/gm) at the discontinuous larval feeding pattern with conc. 0.25%, then by a non-significant increase of *ca*. 14% at the continuous larval feeding pattern (0.85 ± 0.22 mg/gm) when compared to the control mean value in Table 6. Also, a significant or non-significant decrease, by *ca*. 86, 87, and 22%, in the total carbohydrate content, was observed among the applied *L. sativum* concentrations, regardless of whether their larvae were fed continuously or discontinuously on mulberry leaves-enriched with *L. sativum* seed powder. At the larval continuous feeding pattern, the quantified increase in the total carbohydrate content was in a dose-dependent way (0.11 ± 0.04, 0.85 ± 0.22, and then 6.34 ± 0.02 mg/gm); while at the discontinuous feeding pattern, there was no obvious trend for the increase or the decrease in the total carbohydrate content (Table 6). In general, the quantified mean values of the total protein, lipid, and carbohydrate contents were, to a large extent, statistically equal to, and higher or somewhat marginally lower than their corresponding mean values in the control group (Table 6).

The average body weights (± S.E.) of *B. mori* female moths, at death, resulting from *L. sativum* continuously fed larvae ranged between 0.30 ± 0.02 gm and 0.41 ± 0.03 gm; while those resulting from the larval discontinuous feeding pattern ranged from 0.28 ± 0.01 to 0.30 ± 0.02 gm, as compared to the corresponding mean value (± S.E.) of 0.34 ± 0.02 gm in the control group (Table 3). There were no significant differences (α level = 0.01) in female moth average body weights, at death, among both the experimental and the control groups. With this parameter, the observed insignificant increase (18.08%) or decrease (3.69–20.08%) in the experimental groups, over their control value, appears to be a dose-dependent.

In general, apart from *L. sativum* seed powder concentration used in the present study, the findings in Table 7 indicate that enrichment of mulberry leaves with *L. sativum* seed powder significantly increased all mean values of the subject economic parameters of *B. mori*, except for values of both the cocoon shell ratio percentage and the protein content in the female moth internal reproductive tract at death. In the experimental group, the mean values of the former parameter were significantly lower by nearly 18–21% than that of the control group; whereas a decrease, by up to 2.84%, in the mean values of the latter parameter was a marginally significant when compared to the control group (Table 7). On the one hand, there was no significant difference between the two experimental groups, viz., the continuous or the discontinuous larval feeding pattern, in the mean values of the following nine parameters (Table 7): cocoon shell weight, male or female longevity, spermatophore count per female, total protein content in the female moth internal reproductive tract at death, female moth body weight at death, and male body size (measured as forewing length, hind femur length or hind tibia length). On the other hand, there was a significant difference between both of the experimental larval feeding patterns, the continuous and the discontinuous, in mean values of the following eleven parameters (Table 7): five of them were in favour of the former feeding pattern (pupal weight, cocoon weight, egg surface area, egg volume, and total carbohydrate content in the female moth internal reproductive tract at death) and six parameters were in favour of the latter one (cocoon shell ratio%, female fecundity, total lipid content in the female moth internal reproductive tract at death, and female body size as measured by forewing, hind femur or hind tibia lengths).

## Discussion

Many early and recent studies have been focused on the idea of how to nutritionally enrich mulberry leaves, *Morus* spp., with various botanical or animal supplements (e.g., botanical extracts, natural products, organic and inorganic compounds) in order to gain a better yield of *B. mori* silk production and/or egg production^14,15^. Empirically, *B. mori* physiological performance and its consequent outputs, in a form of a good yield of silk or eggs, is greatly influenced by nutrients in its larval diet, where the adult females do not feed^16,17^.

Based on personal observations on *L. sativum* seed powder nutritional value and its phagostimulant, as well as its antimicrobial properties, it was fruitful to nominate such nutritious seeds, as a fine powder, for enriching *B. mori* larval diet, mulberry leaves; hopefully to provide *B. mori* larvae with a more nutritious and economically cheap rearing diet. Chemically, *L. sativum* seed powder is a highly nutritive natural source of proteins, carbohydrates, lipids, vitamins, minerals, mucilage, fibers, steroids, antioxidants, sterols (β-sitosterol), and other essential nutrients or phytochemicals which enable it to be used in the enrichment of several foods and beverages or juices^18,19^. In the literature, there are no available reports on the effect of *L. sativum* seed powder on *B. mori* economic parameters; hence, the present study seems to be the first report in this concern.

As expected, the present findings had shed some light upon the promising role of *L. sativum* seed powder in sericulture. Firstly, obtaining highly fecund *B. mori* female and male moths. Secondly, regardless of the concentration used of *L. sativum* seed powder or the larval feeding pattern, viz., continuous or discontinuous, the female or the male moth reproductive performance was remarkable based on the percent increase over the control in mean values of the following parameters: fecundity (*ca*., 113–179%; or *ca.,* a twofold to threefold increase), egg surface area (*ca*., 23–26%) or egg volume (*ca*., 44–49%), female or male longevity (*ca*., 8% or 29–30%, respectively), spermatophore counts per female (*ca*., 100–104%), and female or male moth body size, measured as forewing, hind femur or hind tibia lengths (*ca*., 5–25% for female moths or 5–10% for male moths). By comparing the effect of *L. sativum*-enriched larval food on *B. mori* egg yield (mean ± S.E.) per female moth in the experimental group (i.e., ranged between 958.41 ± 33.03 and 1255.92 ± 34.19 eggs, and 450.63 ± 17.38 eggs in the control group; see Table 7) and the corresponding mean values which have been reported in the available literature, the present *L. sativum* diet exhibited the best findings. In the literature, *B. mori* larvae which have been fed on mulberry leaves with or without nutritive supplements, the fecundity of their emerged female moths ranged between 329 and 835 eggs for treated leaves or between 220 and 750 eggs for the untreated ones^20,21^.

The chemical composition of *L. sativum* seed powder seems to be the main reason for the present good performance of *B. mori* in terms of cocoon economic parameters and moth reproductive success or fitness parameters. Therefore, a nutritional physiological explanation for the present unusually positive effects of *L. sativum* seed powder on the two vital physiological events, viz., the silk production and the egg production, might be in the following line: As reported by several authors, such important physiological events in *B. mori* life cycle depend on a number of influential factors; nutrition is one of these factors, where it plays an effectual role in sericulture^17,21^.

The percent increase over the control in *B. mori* pupal and adult economic parameters are evidently due to the improvement in the efficiency of *B. mori* larval diet by adding nutritious supplements to mulberry leaves. Although mulberry leaves per se are a complete diet for *B. mori* larvae; however, it is possible to improve the nutritional value of these leaves by enriching them with various inexpensive nutrients in order to enhance *B. mori* larval, pupal, and adult economic parameters. As might be expected, *B. mori* larvae would nutritionally benefit from such a handy enriched food viz*.,* mulberry leaves per se and *L. sativum* seed powder. Hence, the phenomenal increase in the egg yield per *B. mori* female moth could be attributed to the enrichment of mulberry leaves with *L. sativum* seed powder where *B. mori* larvae have fed on a more nutritious food that would be rich in proteins, lipids, carbohydrates, vitamins, minerals, antioxidants, sterols, phospholipids, phytochemicals, etc*.* Thus, *B. mori* female moths obtained from well-nourished larvae laid more eggs (experimental group) than those from, relatively, poorly fed ones (control group). On the one hand, it is known that in the majority of insects, a protein-rich nutrient is the most vital factor affecting the total egg output^20^. On the other hand, protein is important for egg yolk production; where during the early stages of egg development, much protein is ingested and the intake of protein stimulates the corpora allata which secrete a factor leading to increased carbohydrate intake during the period of yolk deposition in the egg^22^. Therefore, these information may explain the remarkable increase in both the egg size and number laid per *B. mori* female moth in the presence of *L. sativum* seed powder in the larval food; as well as the unexpected reasonable increase in the total protein, lipid, or carbohydrate content in the internal reproductive tract of *B. mori* female moths at death; taking into consideration that *B. mori* moths do not feed, and the physiological or energetic costs concomitant with production of large eggs and being highly fecund moths rely heavily on their protein, lipid, and carbohydrate reserves that the female moths acquire through their larval feeding^20,23^. Expectedly, *B. mori* egg size can increase if physiologically important nutrients are at hand. In fact, this is the case in the present study where the enrichment of mulberry leaves with *L. sativum* seed powder seems to be the main cause beyond such desirable expectations, which could be supported by a similar opinion of Wiklund and Karlsson^24^ in this concern. Jones^25^ noted that larger females of the cabbage butterfly, *Pieris rapae* L. were more fecund than smaller ones, as well as the latter females tended to produce smaller eggs; hence, the egg size is dependent on female body size^26,27^. Consequently, the efficiency of *L. sativum* seed powder in *B. mori* larval diet has been translated into the following advantages, from a sericulture point of view, for both *B. mori* male and female moths**:** large body size, highly fecund and large-egged females, and a noticeable reproductive performance or fitness of male moth, in terms of significant increases in spermatophore counts per female moth. Similarly, Fox and Czesak^28^ and Malik and Reddy^29^ reported that better nutrition during larval period can greatly influence the female reproductive investment in both egg size and egg numbers. Thus, the larval diet and the female body size each, to a large extent, seems to have a physiological effect on both egg number and egg size^30,31^. Moreover, the availability of growth promoting compounds in botanicals (e.g., *L. sativum* seeds) may improve the bioavailability of nutrients for digestibility and may provide more energy for reproduction, leading to an increase in egg output of *B. mori*^32,33^. Inclusively, the larval feeding diet should be more important than the body size in determining the egg size^34,35^. On the other hand, in general, the diet responsible for production of greater numbers of eggs also produces large eggs^36^. Meanwhile, Honěk^26^ stated that female body size is a principal constraint on insect potential fecundity. Further, it is commonly assumed that fecundity increases with body size in most animals and with insects this has been shown to be true^35,37^.

Evidently, *L. sativum* seed powder-enriched mulberry leaves confer advantages not only to *B. mori* female moths but also to male moths in terms of an increase, over the control, in body size of *ca.* 5–10%. Also, the present data reveal an increase, over the control, in spermatophore counts per female moth of *ca.* 100–104%. Spermatophore count in *B. mori* dissected female moths seems to be useful in estimating the number of matings^38^ per female moth. Findings of this study show that *B. mori*-female moth fecundity increases significantly, in the experiment group, with the number of spermatophores received (i.e., on average, *ca.* three spermatophores per female moth, as compared to nearly one spermatophore in the control moths). Papers of Eady et al.^39^ and Arnqvist and Nilsson^40^ could support the present results as they demonstrated that in many insects, mating frequency enhances female fecundity, as well as female that copulating repeatedly with the same male lay more eggs. The latter observation is congruent to the present case. Thus, both mating frequency and large body size are seemed to be important for *B. mori* male or female reproductive success. These observations are in agreement with Honěk^26^ and Teng and Zhang^41^ who also reported similar findings in their experimental lepidopterous species. From the literature, it has been shown that an insect body size is not the only parameter influencing male acceptance by female during courtship; meanwhile, a large body size should enhance male reproductive fitness or performance^42^. Also, the production of sex pheromone(s) released by male moths, that may facilitate female acceptance during courtship, oftenly requires precursors, e.g., alkaloids^43^. These precursors are present in plants^44^ and insect male moths obtained these alkaloids through larval feeding^45,46^. Since *L. sativum* seed powder mainly contains alkaloids^47,48^ therefore, the findings reported here may boost up the positive influential role of *L. sativum* seed powder in *B. mori* larval food. It seems that the male larval nutrition positively affects the reproductive fitness of both sexes of *B. mori*; taking into consideration the findings of Eisner and Meinwald^49^ and LaMunyon and Eisner^50^ that larger insect males provide greater amounts of alkaloids and nutrients that might both be good indicators of male and female fitness or reproductive success. However, a further study is certainly required to confirm the exact role of *L. sativum-*seed alkaloids as precursors of a male sex pheromone(s).

In addition to the observed remarkable increases in reproductive success or fitness parameters of *B. mori* male or female moths associated with their larval feeding on *L. sativum* seeds powder-enriched mulberry leaves, another bright side of this botanical additive relates to *B. mori* pupal economic parameters has been observed as well. The silk productivity, as measured by sericulture economic parameters like cocoon weight, cocoon shell weight, and pupal weight, showed significant increases in their mean values over the controls. Irrespective of *L. sativum* seed powder concentration used or *B. mori* larval feeding pattern, viz., continuous or discontinuous, the cocoon weight, the cocoon shell weight, and the pupal weight had increased significantly by a percent of nearly 30–38%, 7–10%, and 39–50%, respectively. However, the cocoon shell ratio (%) was higher by about 18–21% in the control group than in the experimental group. The latter result was expected and could be referred to the above-mentioned significant increase in pupal weights observed among *L. sativum*-fed group as compared to the corresponding significantly low weights in the control group. This finding is in agreement with those of Trivedy et al.^51^ and Saad et al.^52^ but with other nutritious additives. The present improvement in *B. mori* cocoon economic parameters could be attributed to the enrichment of mulberry leaves with extra nutrients which are amply available in *L. sativum* seed powder. Results of Gokavi et al.^53^ and Al-Snafi^48^ could boost the present findings as follows**:** these authors mentioned that the most abundant amino acid in *L. sativum* seeds is glutamic acid (*ca*., 19–24%) followed by aspartic acid (*ca*.,10–12%) then glycine, alanine, and serine acids (each *ca*., 4–6%). On the other hand, *B. mori*-larval feeding on mulberry leaves with high amounts of proteins and amino acids leads to turnover of proteins and amino acids which are concomitant with a high increase in glutamic and aspartic acids. Such amino acids are necessarily required for silk synthesis; not to mention that silk proteins of the cocoon shell (i.e., fibroin and sericin) are made up of polypeptide chain of amino acids, specifically alanine, glycine, and sericin. These amino acids are probably assimilated by *B. mori* larvae when they feed on protein-rich food^54,55^. Also, according to Murugan et al.^56^, higher levels of protein and the amino acid serine in *B. mori* silk glands are observed after the larval feeding on mulberry leaves enriched with botanical extracts (e.g., *L. sativum* seed powder) which are rich in proteins, amine acids, carbohydrates, minerals, vitamins, phytochemicals etc. Knowing that the fifth-larval-instar of *B. mori* utilizes about 65% of the absorbed nitrogen, therefore, nitrogenous compounds present in the larval feeding diet can have a great effect on larval growth and cocoon production^57,58^. Meanwhile, Lu and Jiang^59^ have stated that *B. mori* larvae utilize 72–86% of amino acids from mulberry leaves, and more than a 60% of the absorbed amount is utilized for silk production. The authors added that absorption of amino acids in midgut of *B. mori* larvae depends on potassium ion. Additionally, the enrichment of mulberry leaves with the non-essential amino acids, aspartic (1 and 2%), alanine (0.5%), glycine (0.5%) or glutamic (1%) leads to increases in larval weight (by about 10–14%), silkworm economic parameters, larval food consumption, and absorption percentage^58,60^. Hence, amino acids are greatly important for the silk filament synthesis and could also improve the silk yield if added in adequate quantities^61^.* L. sativum* seeds are also a good source of vitamins, minerals, and antioxidants (due to their high content of phenolic compounds)^19,48^. Moreover, *L. sativum* seeds are rich in vitamins, C (ascorbic acid), A (β-carotene, provitamin A), E (tocopherols), and B vitamins such as thiamine (B_1_), riboflavin (B_2_), and nicotinic acid (niacin, B_3_)^18,62^. Many reports have also stated that magnesium, calcium, phosphorus, potassium, iron, manganese, and zinc (amply present in *L. sativum* seeds^62,63^) are essential elements required by *B.mori* to stimulate the metabolic activity^64^; to improve the quality of the silk thread and cocoon parameters as well^65^. On the other hand, *B. mori* larvae which fed on a diet containing more minerals, divert minimum energy for maintenance; hence, the larvae can channel maximum energy for silk production^17,66^.

Vitamins and minerals, as other nutritive additives to mulberry leaves, play an important role in *B. mori* larval nutrition and hence increase the sericultural yield^61,67^. Since *B. mori*-fifth-larval-instar is the most important instar of silk production^68^, therefore, Ito^69^ and Horie^70^ mentioned that many members of vitamin B-complex, such as thiamine (B1), riboflavin (B2), and niacin (B3) have a direct or an indirect influential role on some economic parameters of *B. mori* as follows: Thiamine is important for metabolism and significantly increases cocoon weight, cocoon shell weight, and female moth fecundity; riboflavin improves the silk production, and niacin is important for releasing energy from carbohydrates and lipids; it is also important in protein metabolism, and production of some hormones. Furthermore, vitamin C has been reported to enhance remarkably *B. mori* silk yield^67,71^. Besides, it acts as a phagostimulant or a gustatory stimulant^69,72^. The natural antioxidants like tocopherols (vitamin E), phytosterols, and carotenoids, which are available in *L. sativum* seeds^19,73^, have some growth promoting effects on *B. mori* larvae which result in increased silk output as reflected by improvement in cocoon parameters, i.e., cocoon weight, cocoon shell weight, and pupal weight^69^; also have phagostimulant properties such as sterols^74^ and their principal role in *B. mori* larvae seems to be nutritional^75–77^. Further, there is a possibility of conversion of sterols, in *B. mori* larval food, into active hormonal substances by *B. mori* larvae^77^. Also, *B. mori*-larval feeding diet's efficiency is highly increased in the presence of phospholipids^75,77^. *L. sativum* seeds are an excellent source of phospholipids (5.8gm per 100gm seeds^53^).

In summary, apart from the cocoon shell ratio (%), the present improvements in sericulture different parameters observed in the experimental group against the control group might be due to the nutritionally and physiologically important phytochemicals present in *L. sativum* seed powder, which proved to be essential for *B. mori* in addition to their natural food, mulberry leaves. The review of literature has revealed that these phytochemicals could act as a physiological stimulator of protein synthesis by tuning or stimulating the neuro-endocrine system in *B. mori* larvae and ultimately enhance the protein synthesis^56,78^ which promotes both the silk synthesis, as evidenced by the significant increases in the cocoon weight, the cocoon shell weight, and the pupal weight^17,55^, and the egg yield of the silkmoth^20,22^. Also, these phytochemicals could act efficiently as follows: (1) Phagostimulant (i.e., may increase the larval appetite leading to an increase in *B. mori* silk and egg productivity^7,77^; (2) Antioxidant (i.e., may increase the absorption of the mutrients in the larval midgut leading to a high larval growth and consequently an increase in *B. mori* economic parameters^72,79^; (3) Promoter of larval growth and silk production (i.e., due to their physiological stimulation^80,81^; (4) Antimicrobial agent (i.e., for achieving a better rearing and productivity by avoiding *B. mori*-diseases which badly affect their health and productivity^6,82^. There is only one published paper in the literature which denoted with the positive effect of *L. sativum* seed oil on *B. mori*, where this oil had increased *B. mori* productive characters and can be utilized as an additive to increase the silk yield^83^. Based on all the above-mentioned findings, the present study or the corresponding ones in the literature, the phenomenal egg yield and the improvement in the cocoon economic parameters logically might be due to the nutritionally or physiologically valuable biochemical components amply available in *L. sativam* seed powder. These results are in agreement with the findings of many authors but with botanicals other than the garden cress, *L. sativum* seed powder^81,84^.

In conclusion, the authors believe that the present study is the first to evaluate the efficacy of *L. sativum* seed powder as a promising food additive for improving the nutritional value of mulberry leaves and consequently enhancing the economic parameters of *B. mori*; hopefully to gain ground in the sericulture industry, hence increasing the national revenue. The ANOVA indicates that apart from *B. mori* shell ratio (%) in the experimental group, *B. mori* larval feeding on *L. sativum*-enriched mulberry leaves significantly increased all mean values of the different economic parameters evaluated in the experimental group comparing with their corresponding values in the control group. In general, the present study indicates that *B. mori* moths, which do not feed, are efficiently able to utilize the stored physiological resources or reserves (i.e., protein, lipid, and carbohydrate) gathered during the larval stage and remarkably enhanced the reproductive output or performance of their male or female moths. The latter parameter is translated into phenomenal high egg counts, high spermatophore counts, large eggs, large body size, and, in general, a reasonable content of protein, lipid, and carbohydrate in the internal reproductive tract of *B. mori* female moths at death. Hence, as being a good natural source of protein, lipid, and carbohydrate, *B. mori* female moth bodies at death could be nominated as a raw material in certain manufactures like pet feed formulations instead of discarding them as a source of environmental pollution. On the other hand, the enrichment efficacy of mulberry leaves with *L. sativum* seed powder is also translated into significant increases in *B. mori* cocoon economic parameters such as cocoon weight, pupal weight, and cocoon shell weight. Consequently, in sericulture field, great expectations of *L. sativum* seed powder seem to be promising.

### Supplementary Information

Supplementary Information 1.

Supplementary Information 2.

Supplementary Information 3.

Supplementary Information 4.

Supplementary Information 5.

Supplementary Information 6.

Supplementary Information 7.

Supplementary Information 8.

Supplementary Information 9.

Supplementary Information 10.

## Data Availability

Data generated or analysed during this study are included in this published article [and its supplementary information files].
