# Supplementary figures and images for: Effects of mulberry leaf enrichment with Lepidium sativum L. seed powder suspension on the economic parameters of Bombyx mori L
Source: Sci Rep. 2024 Aug 23;14:19600. doi: 10.1038/s41598-024-67128-0 (PMC11343774; doi:10.1038/s41598-024-67128-0)

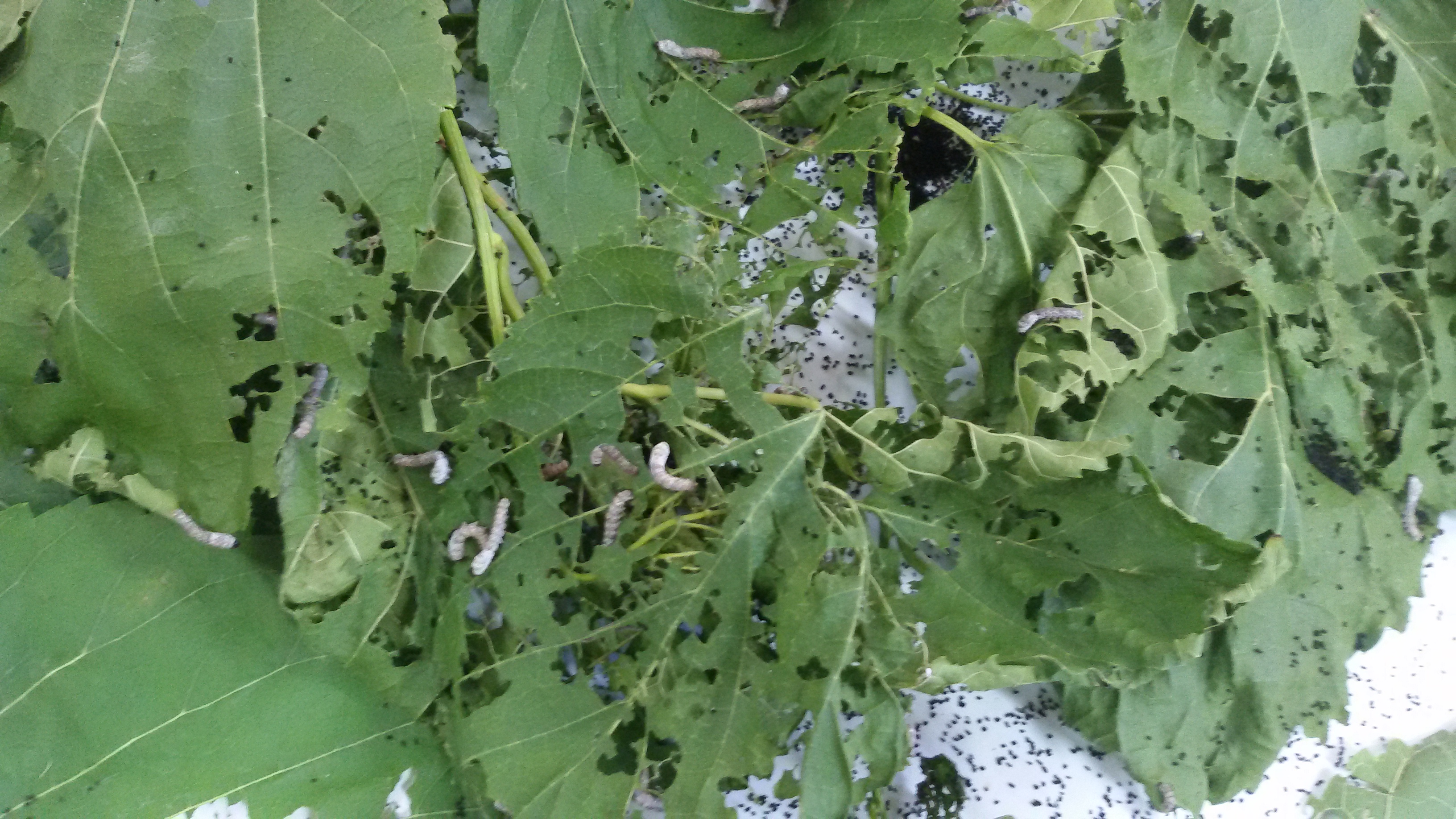

Supplement: Supplementary file 1 — Supplementary Information 1. [file 41598_2024_67128_MOESM1_ESM.jpg]

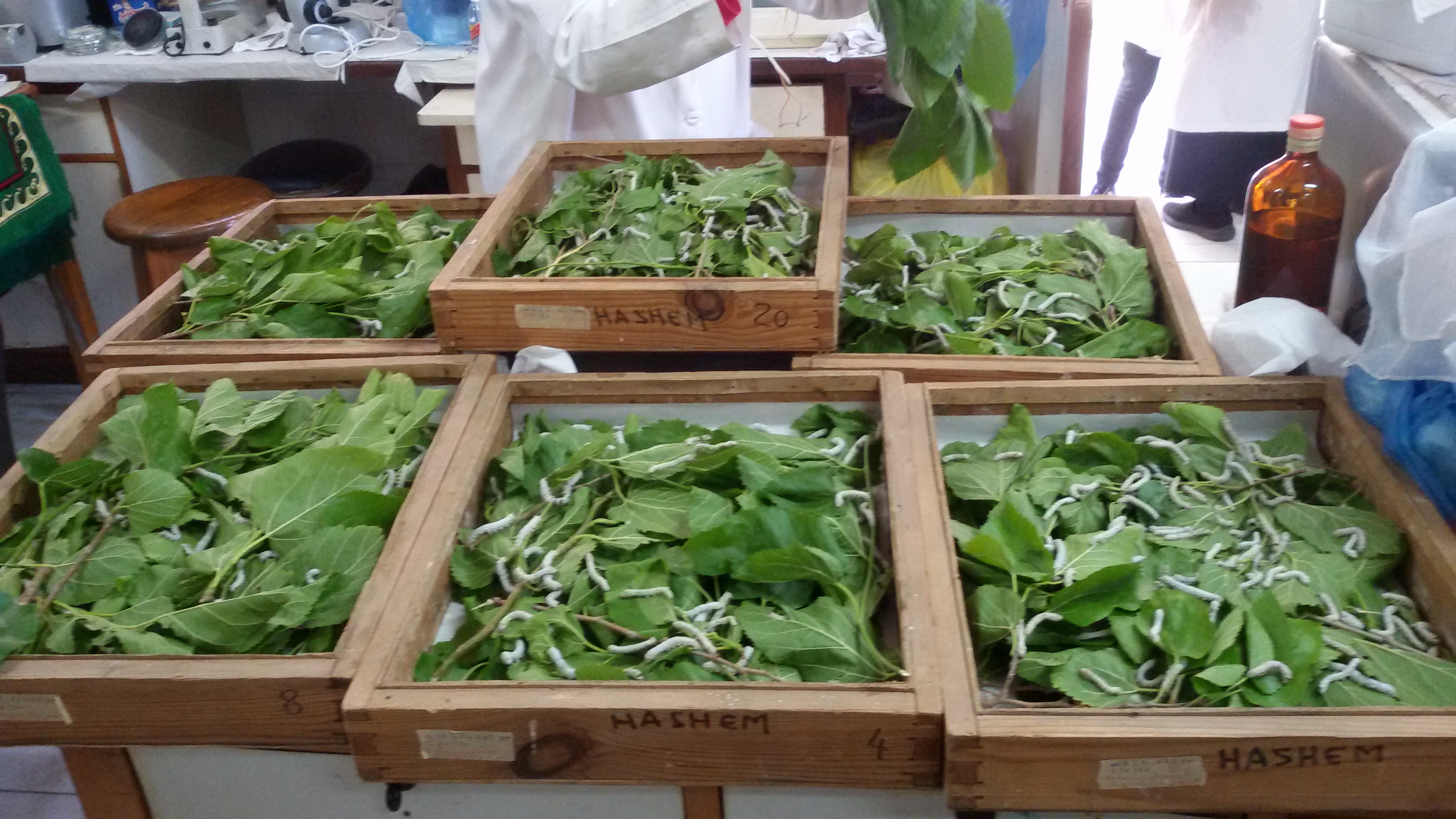

Supplement: Supplementary file 2 — Supplementary Information 2. [file 41598_2024_67128_MOESM2_ESM.jpg]

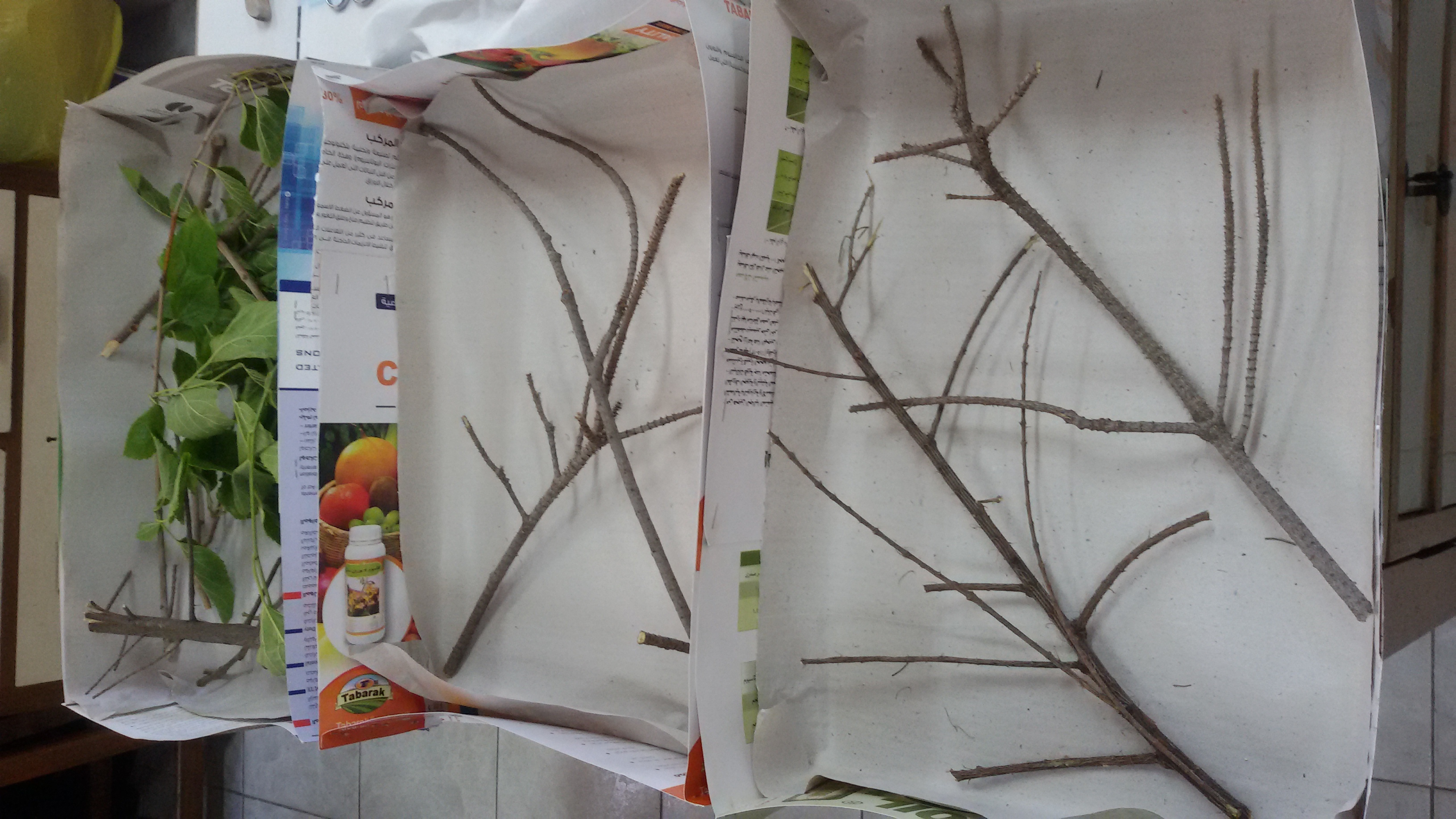

Supplement: Supplementary file 3 — Supplementary Information 3. [file 41598_2024_67128_MOESM3_ESM.jpg]

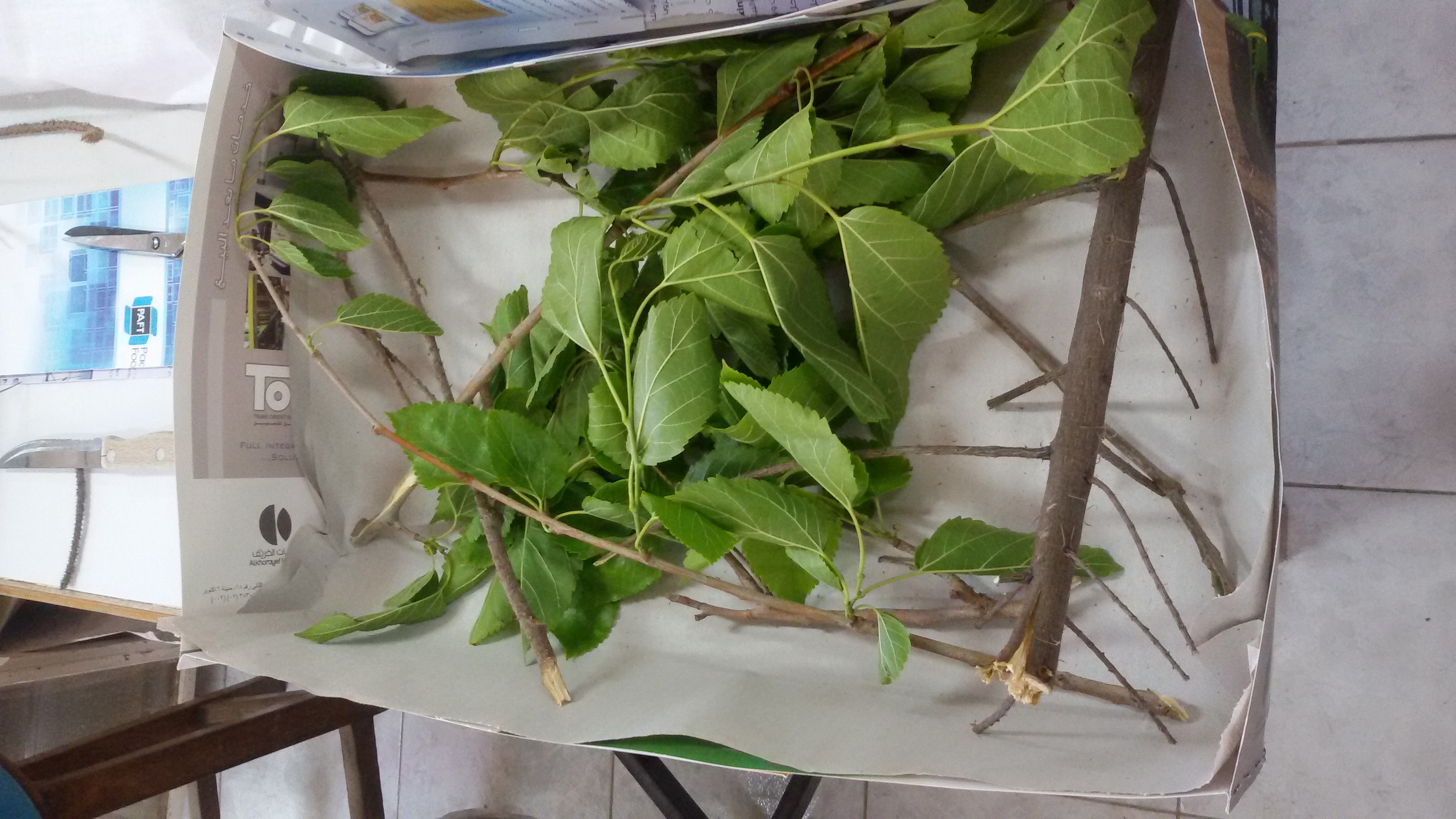

Supplement: Supplementary file 4 — Supplementary Information 4. [file 41598_2024_67128_MOESM4_ESM.jpg]

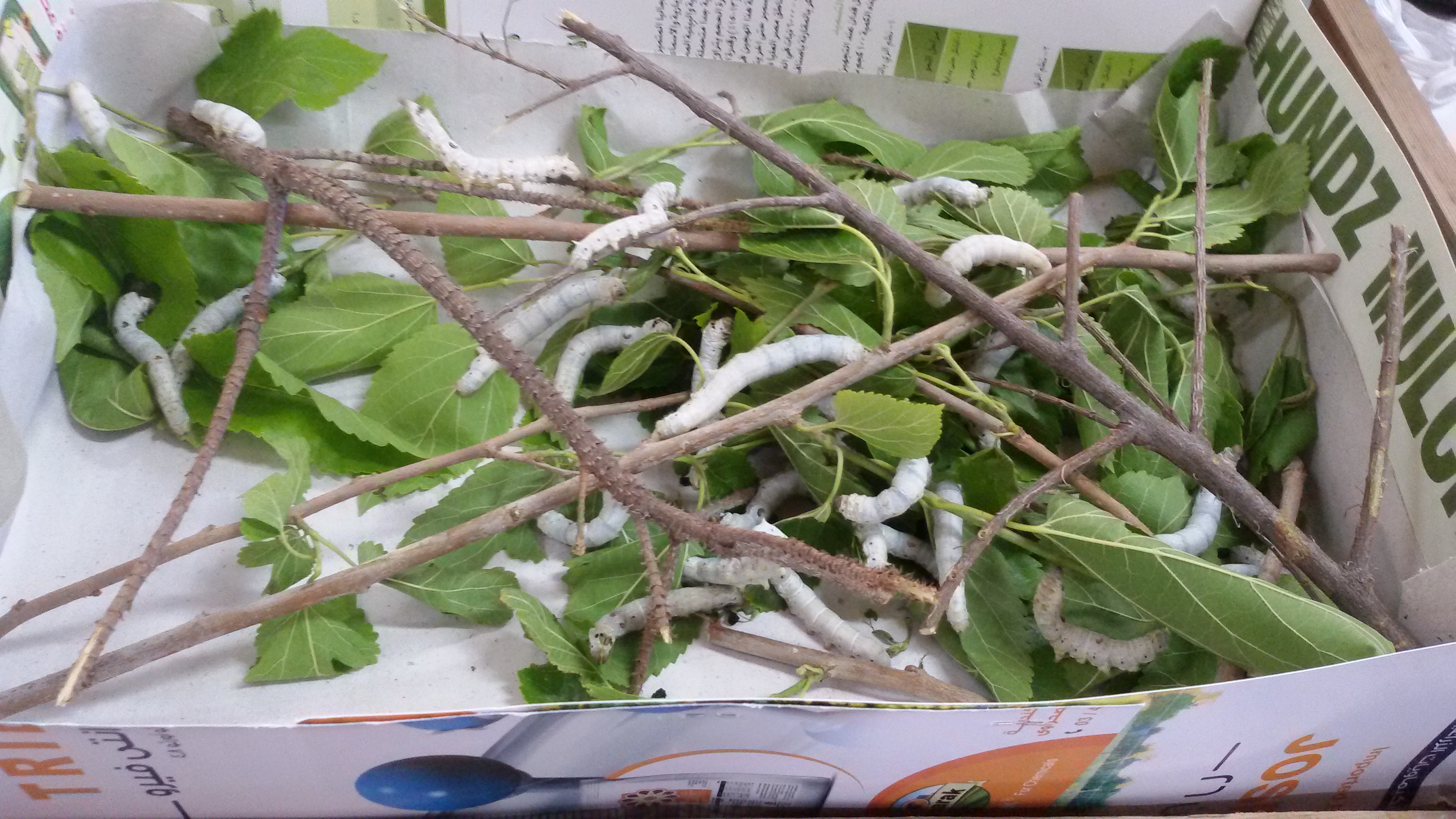

Supplement: Supplementary file 5 — Supplementary Information 5. [file 41598_2024_67128_MOESM5_ESM.jpg]

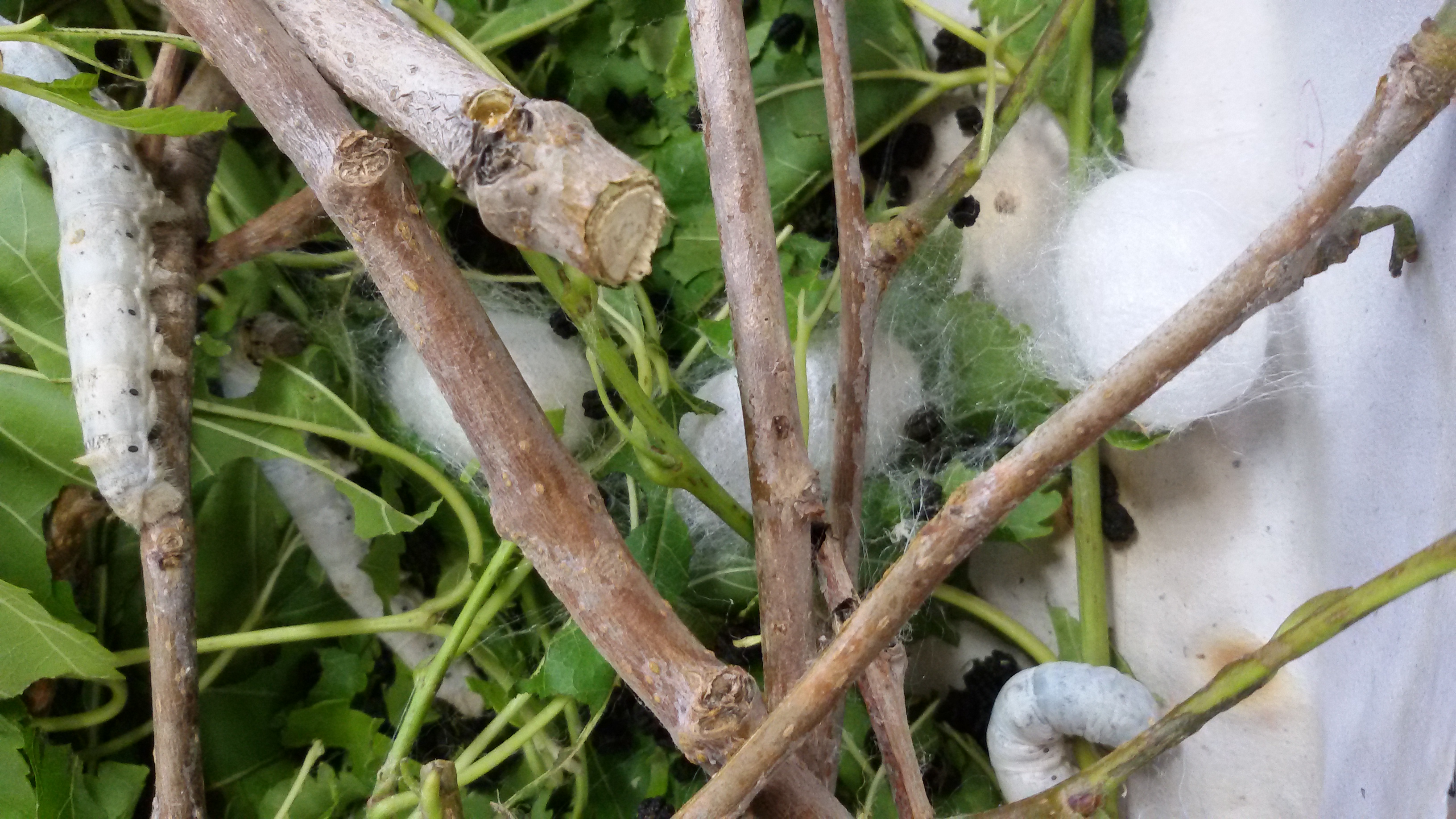

Supplement: Supplementary file 6 — Supplementary Information 6. [file 41598_2024_67128_MOESM6_ESM.jpg]

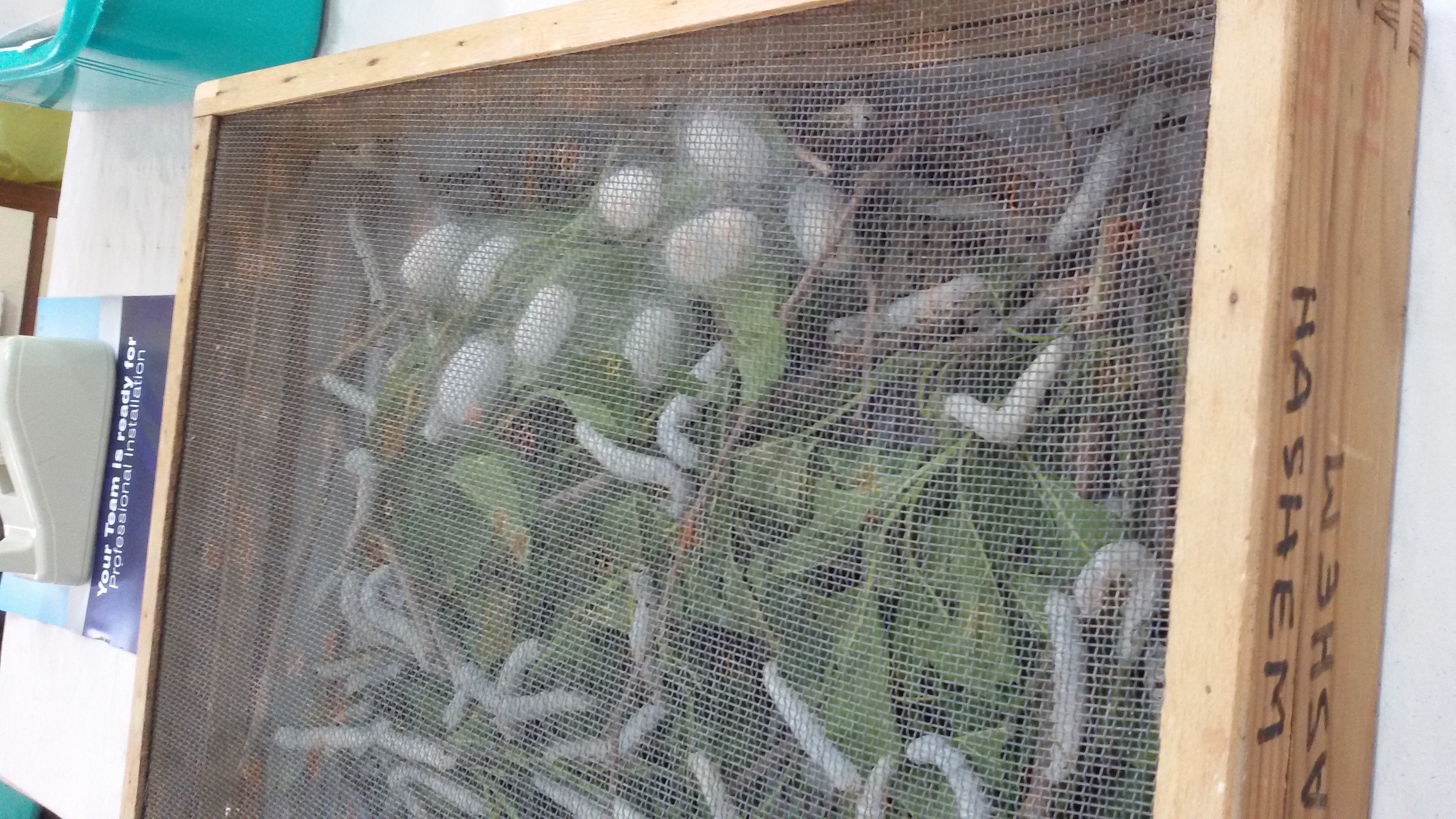

Supplement: Supplementary file 7 — Supplementary Information 7. [file 41598_2024_67128_MOESM7_ESM.jpg]

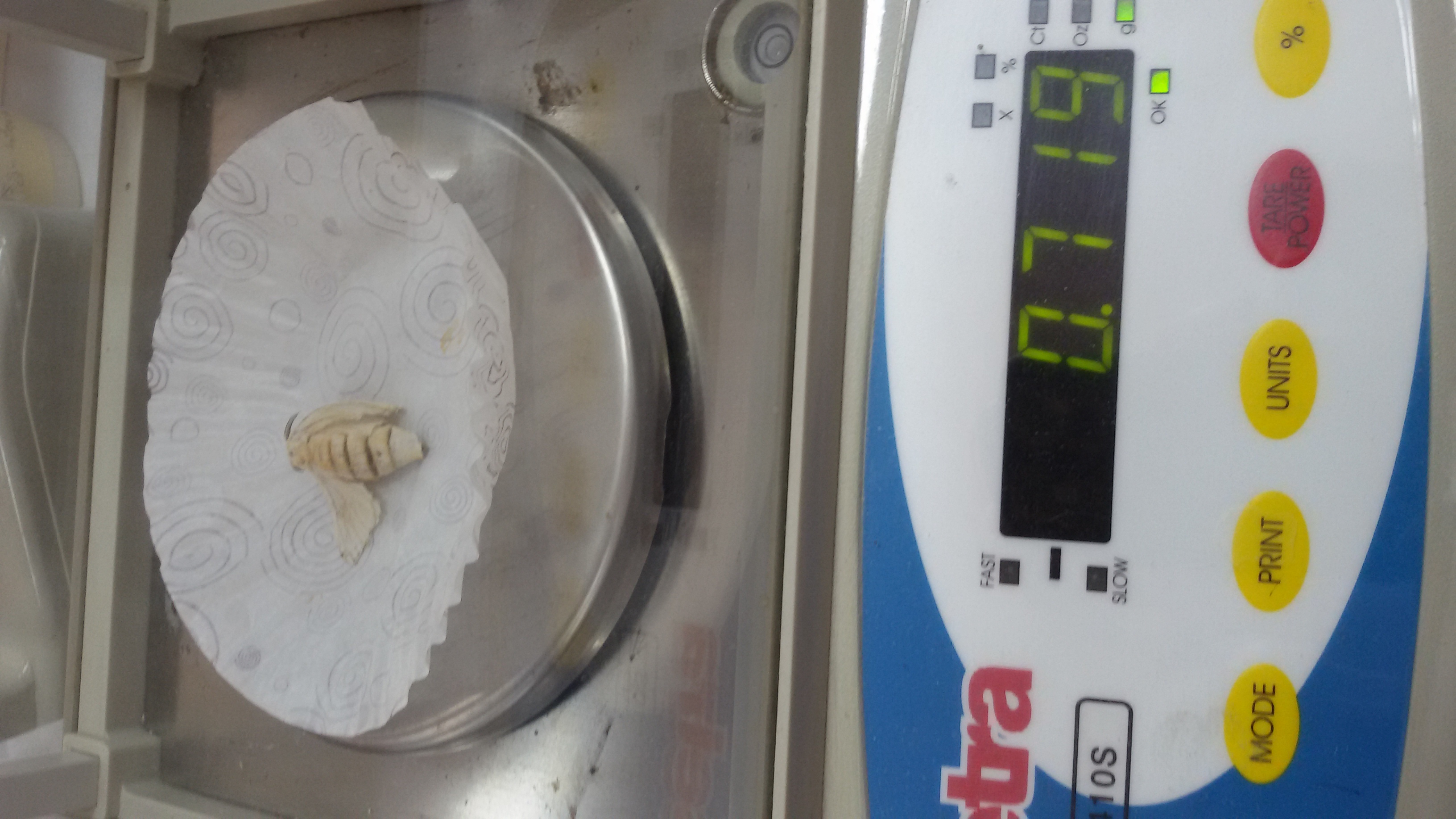

Supplement: Supplementary file 8 — Supplementary Information 8. [file 41598_2024_67128_MOESM8_ESM.jpg]

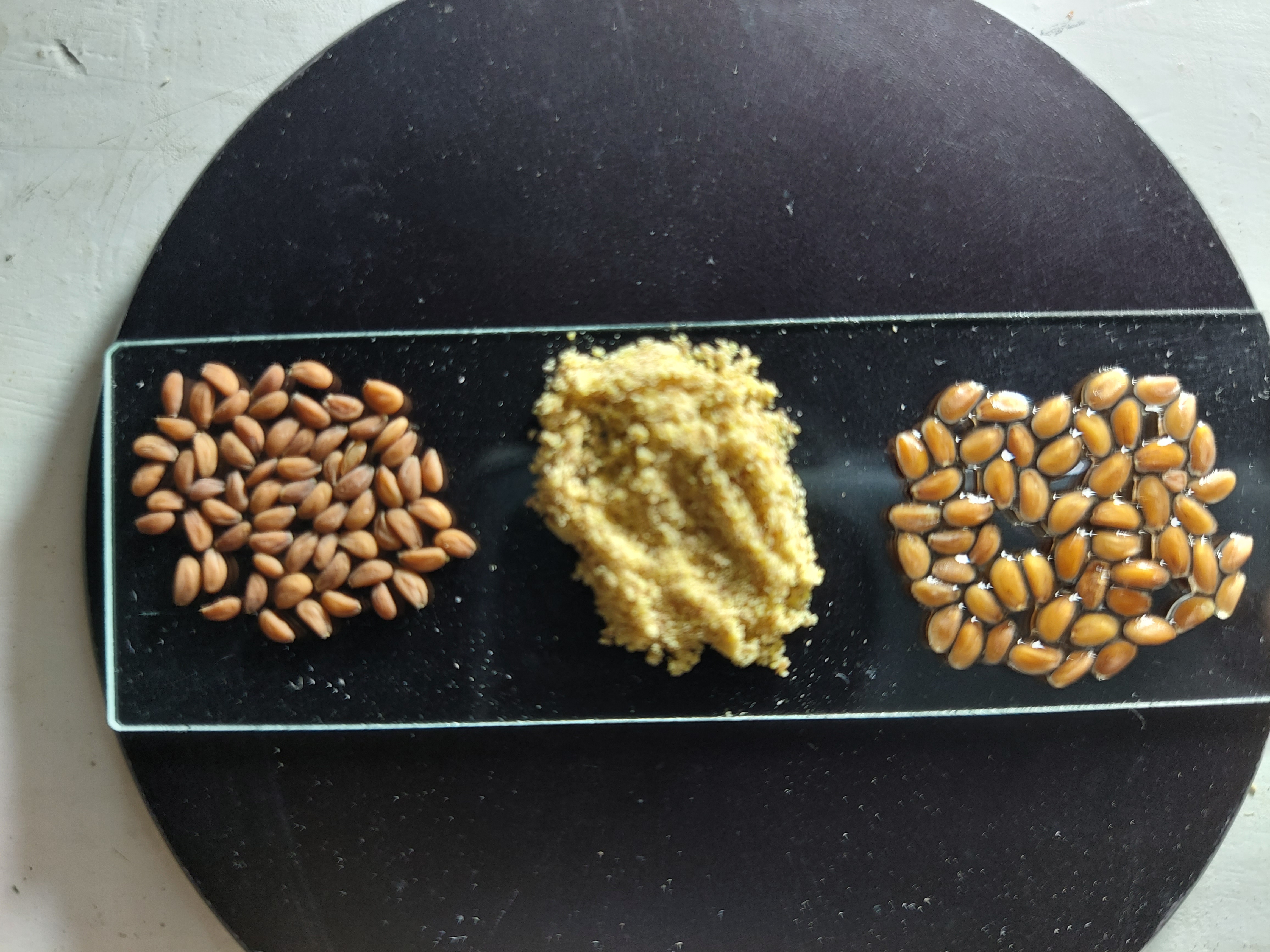

Supplement: Supplementary file 9 — Supplementary Information 9. [file 41598_2024_67128_MOESM9_ESM.jpg]
